# Translational and Clinical Significance of DAMPs, PAMPs, and PRRs in Trauma-induced Inflammation

**DOI:** 10.26502/acbr.50170279

**Published:** 2022-08-26

**Authors:** Vikrant Rai, Gillian Mathews, Devendra K Agrawal

**Affiliations:** Department of Translational Research, Western University of Health Sciences, Pomona, CA 91766, USA

**Keywords:** Biomarkers, DAMPs, Inflammation, Multiple Organ Failure, PAMPs, SIRS, Trauma

## Abstract

Increased morbidity and mortality after polytrauma due to multiple organ failure (MOF) is a major concern for clinicians. Systemic inflammatory response syndrome (SIRS) and sepsis are the major underlying causes. Damage-associated molecular proteins (DAMPs) released after polytrauma induce an inflammatory immune response to repair the tissue, however, persistent inflammation finally results in immunosuppression and MOF. During immunosuppression, additional exposure of the traumatized tissue to pattern-associated molecular patterns (PAMPs) further adds to the continuum of inflammatory cascade causing sepsis. These two hits worsen the condition of the patient and increase morbidity and mortality. Thus, it is critical to stratify the patient based on trauma severity and inflammatory biomarkers levels and design treatment accordingly for a better clinical outcome. Although some of the molecular mechanisms involved in SIRS and MOF after polytrauma have been reported, there is limited information on the critical factors related to the study of DAMPs and PAMPs, including the timing of sampling (time elapsed after trauma), source of sampling (blood, urine, saliva), proteomics and metabolomics, multiplex plasma assay, comparative interpretation of the results from various sources and diagnostic value, and interpretation on the translational and clinical significance. Additionally, there is limited literature on DAMPs like heat shock proteins, mitochondrial DNA, neutrophil extracellular traps, and their role in SIRS and MOF. Further, it is also important to distinguish between the biomarkers of SIRS and sepsis in a time-bound window to have a better clinical outcome. This critical review focuses on these aspects to provide comprehensive information and thought-provoking discussion to design future investigation and clinical trials.

## 1. Introduction

**Figure 1:**
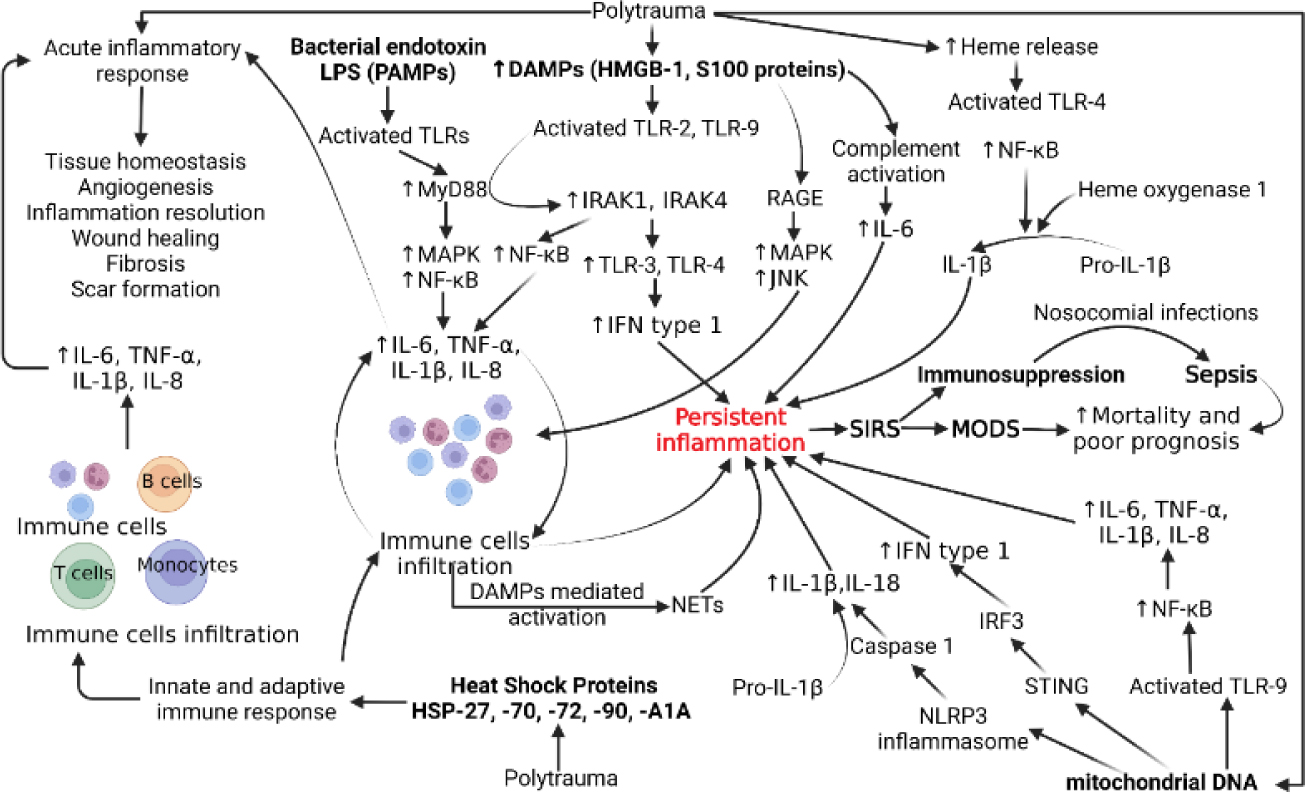
Molecular pathogenesis of systemic inflammatory response syndrome (SIRS), sepsis, and multiple organ dysfunction syndrome (MODS) after polytrauma. Abbreviations: Interleukins (IL), tumor necrosis factor (TNF)-α, damage-associated molecular proteins (DAMPs), pathogen-associated molecular proteins (PAMPs), toll-like receptors (TLRs), receptor for advanced glycation end products (RAGE), lipopolysaccharides (LPS), high mobility group box protein (HMGB)-1, nuclear factor kappa beta (NF-κB), myeloid differentiation primary response 88 (MyD88), mitogen-activated protein kinases (MAPKs-JNK, ERK, and p38), interleukin-1 receptor-associated kinase (IRAK), interferon (IFN), heat shock proteins (HSPs), neutrophil extracellular traps (NETs), NOD-, LRR- and pyrin domain-containing protein 3 (NLRP3), IFN regulatory factor 3 (IRF3), and stimulator of interferon genes (STING).

Trauma accounts for 10% of deaths and 16% of disabilities in the world [1]. In addition to the initial injury, trauma is further complicated by trauma-induced inflammation that can progress to systemic immune response syndrome (SIRS), sepsis, and multi-organ failure. As a result, trauma-induced inflammation has become one of the leading causes of death in patients between the age of 1 and 46 years. Prior to 1980s, sepsis and SIRS were believed to be solely caused by products of microbial pathogens called PAMPs (pathogen-associated molecular patterns) [2–4]. Since then, research has shown that SIRS can occur in the presence of infection or sterile tissue injury. Accordingly, the former is now recognized with sepsis as non-sterile trauma-induced inflammation and the latter is recognized as sterile trauma-induced inflammation. Trauma induces the release of sterile molecules called damage-associated molecular patterns (DAMPs). Examples of DAMPs include mitochondrial DNA (mtDNA), histones, high mobility group box protein −1 (HMGB-1), S100 proteins, and heat shock proteins which are released from immune cells and induce an inflammatory response. DAMPs further activate the immune response by triggering the classical pathway, upregulating the production of C3a and C3b, resulting in a release of inflammatory cytokines that upregulate sterile inflammation and the formation of the membrane attack complex (MAC). The release of inflammatory cytokines increases the permeability of endothelium which further increases the ability of DAMPs and other inflammatory mediators to access the intracellular space. The disease process of trauma-induced inflammation is further complicated by a compensatory release of anti-inflammatory molecules, therefore, increasing patient susceptibility to infection and sepsis [1, 5–9]. The role of various DAMPs, PAMPs, and PRRs and their signaling pathways have been depicted in Figure 1.

Morbidity and mortality of trauma-induced inflammation have remained high despite medical advancements [3, 4]. This may be due to a clinical difficulty differentiating between SIRS and sepsis. These two conditions can present similarly as they both result from robust activation of the immune system. Unfortunately, their treatments greatly differ and require early intervention to maximize survival. This clinical issue warrants more research into these conditions, specifically into the presence and role of their inflammatory and anti-inflammatory markers as they may be the key to early clinical differentiation, development of novel treatments, and reducing morbidity and mortality. This review critically reviewed the mediators of sterile inflammation and biomarkers that may prove to have therapeutic benefits.

## 2. Mediators of Inflammation after Trauma

A systemic immune response induced after a traumatic injury comprises both innate and adaptive immune arms of the body involving the recruitment of immune cells and production of cytokines like interleukin (IL)-6, IL-1, IL-8, and tumor necrosis factor (TNF)-α. Trauma is also associated with secretion of “self” damage-associated molecular proteins (DAMPs), exposure of the traumatized part of the body to “non-self” pattern associated molecular patterns (PAMPs), activation of various surface inflammatory receptors, and deregulated function of various cells in the body [10, 11]. DAMPs like high mobility group-box protein (HMGB)-1 produced after cell death and S100 proteins secreted from infiltrating neutrophils and macrophages activate the downstream signaling involving receptors for advanced glycation end products (RAGE) and pattern recognition receptors (PRRs) like toll-like receptors (TLRs) including TLR-2, TLR-3, TLR-4, and TLR-9 inducing a systemic inflammatory response by increased secretion of inflammatory cytokines [8, 12, 13] (Figure 1). This systemic inflammatory response mediated by DAMPs after trauma is termed sterile systemic inflammatory response syndrome (SIRS). Along with HMGB-1 and S100 proteins, mitochondrial (mt)DNA and cellular DNA, heme released from RBC lysis, matricryptins, cold-inducible RNA-binding protein, and heat-shock proteins are other DAMPs released after traumatic injury, and levels of DAMPs are directly associated with the level of trauma and inversely related to the clinical outcome [8, 11, 14].

While the exposure of the tissue to PAMPs like lipopolysaccharides (LPS) of gram-negative bacteria, lipoteichoic acids (LTA) of gram-positive bacteria, lipoproteins generated from bacterial cell wall proteins, peptidoglycan, lipoarabinomannan of mycobacteria, double-stranded RNA (dsRNA) produced by viruses, and β-glucans and mannans from fungal cell walls cause sepsis post-trauma [10, 11, 15–17]. SIRS is accompanied by increased oxidative stress, increased secretion of pro-inflammatory cytokines, immune cell infiltration, and complement activation which ultimately result in multiple organ failure (MOF) or multiorgan dysfunction syndrome (MODS), a major cause of mortality [18]. Early inflammatory response mediated by DAMPs leading to SIRS cause organ dysfunction while in later stages synergistic action of DAMPs with PAMPs, infections, embolism, transfusion, surgical complications, and complications associated with prolonged ventilation led to compensatory anti-inflammatory response syndrome (CARS) and MODS. Persistent inflammation immunosuppression catabolism syndrome (PICS) may be another complication due to prolonged stay in intensive care units after severe trauma [3, 13, 19–21]. In the initial phase, acute inflammation is a host-defense mechanism for tissue repair, but persistent inflammation may lead to organ damage and failure. This section focuses on the role of DAMPs, PAMPs, and PRRs in SIRS after trauma.

## 3. DAMPs, SIRS, and Trauma

### 3.1 HMGB-1

In addition to the DAMPs, also called alarmins, released from cell death or necrosis after trauma, DAMPs like HMGB-1, ATP, histones, and mitochondrial DNA may also be released from adipose tissue, especially in the presence of increased levels of pro-inflammatory cytokines [10]. This suggests that obesity may also affect the clinical outcome by altering the secretion of DAMPs and pro-inflammatory cytokines after trauma and higher expression is associated with a poor prognosis. HMGB-1 secreted from adipose tissue, through its downstream signaling, mediates increased secretion of inflammatory cytokines causing increased immune cell recruitment and ultimately organ damage even at a distant site. The release of DAMPs at an early stage of trauma mediates SIRS and causes multiorgan failure. HMGB-1 exert its inflammatory effects not only by stimulating downstream signaling involving RAGE, TLRs, and triggering receptor expressed on myeloid cells (TREMs) but also synergistically with increased expression of reactive oxygen species, acting as a chemotactic agent to recruit immune cells, and as a cofactor for LPS and nuclear DNA increasing the secretion of inflammatory cytokines [13, 22] (Figure 1). DAMPs released after trauma are also involved in the induction of protein expression of adhesion markers on endothelial cells facilitating leukocyte migration and promoting extravasation of immune cells into the injured tissue [11]. Additionally, epithelial cells, fibroblasts, T-cells, B-cells, monocytes/macrophages, dendritic cells, mast cells, natural killer (NK) cells, and eosinophils are also involved in sensing the DAMPs and inducing sterile inflammation [23]. Since these cells are involved in tissue injury of various organs, the interaction between DAMPs and these cells may have an implication for polytrauma and SIRS.

HMGB-1 induces its biological effect through several molecules, including RAGE, TLRs, TREM-1, and S100 proteins playing a role in various inflammatory diseases [12, 24], and the role of RAGE in the induction of sterile inflammation has been reported in polytrauma patients during the later phase [25]. A study [25] reported a decreased expression of RAGE on monocytes and the number of RAGE-positive monocytes after the trauma but increased soluble RAGE (sRAGE). Although there were no significant changes in the expression of HMGB-1 after the trauma, the expression of S100A8 and S100A12 peaked by day 4. The study concluded that IL-6, sRAGE, and methylglyoxal were present early after trauma, and leukocytes, S100A8, S100A12, and AGE-modified proteins peaked at later time points providing evidence for a secondary release of RAGE ligands. These findings supported the role of RAGE in the pathogenesis during early as well as late-stage after polytrauma and targeting the HMGB1-RAGE axis will have therapeutic efficacy in polytrauma (Figure 1). This notion is supported by fewer detrimental pro-inflammatory macrophages while increased anti-inflammatory immune cells after injury by inhibiting the HMGB1-RAGE axis [26]. But it should be noted that the timing of intervention is important and must be considered while treating polytrauma patients.

The release of HMGB-1 from the tissue under stress may also be controlled by other factors. Nrf2 is one such factor and an increased HMGB-1 expression during the first 6 hours followed by a decline associated with lower levels of Nrf2 in early hours and then increased levels at later time points suggest that HMGB-1 and Nrf2 play a concerted role in inducing inflammation in polytrauma patients and that HMGB-1 secretion is regulated by Nrf2 via modulation of ROS levels [27, 28]. These findings suggest that along with HMGB-1, Nrf2 may also be a potential therapeutic target to attenuate chronic inflammation and MODS in polytrauma patients. The notion of targeting HMGB-1 and Nrf2 in polytrauma patients is supported by the results from other studies documenting the regulation of various processes after cell injuries like ferroptosis and apoptosis via an interaction between HMGB-1 and Nrf2 [29, 30].

### 3.2 S100 Proteins

S100 proteins are other DAMPs whose secretion is increased after trauma. S100B levels are biomarkers for brain injury. Seidenfaden et al. [31] reported that S100B concentrations may peak much earlier in traumatic brain injury patients and estimating S100B lesions may help decide the need for other imaging investigations. Moreover, it has also been reported that S100B expression levels are useful as biomarkers not only in traumatic brain injury but also in other types of polytrauma and may be an important predicting factor for survival after polytrauma [32]. Pfortmueller et al. [32] reported an association of higher concentrations of S100B with higher mortality after polytrauma with and without head trauma as there were no significant differences between the two groups. The negative correlation of S100B with survival supported the significance of S100B as a biomarker for survival in polytrauma in addition to head injuries. Similar findings of the prognostic importance of S100B in polytrauma patients were reported by Dang et al. [33]. The study reported a positive association of circulating S100B concentrations with injury severity with higher levels of S100B in severe trauma patients compared to moderate trauma group and higher levels in fatal cases compared to survivors.

The regulation of innate immune response through S100 proteins is also mediated by regulating macrophage inflammation. S100 proteins regulate cell migration and differentiation and since macrophages infiltrate at the site of injury after trauma, persistently increased S100 proteins might be detrimental due to the presence of inflammatory macrophages [34–36]. Another study [37] also supported the fact that S100B levels are not only a biomarker of brain injury but also for fractures and thoracic injuries. Increased levels of S100B are associated with fractures and thoracic injuries after polytrauma. It was also documented that head injury contributes minimally to increased levels of S100B; S100B levels are associated with trauma severity; and normal S100B levels are a good predictor of a positive outcome. To note, the conclusion of [37] regarding the minimal role of brain injury in S100B levels might be because S100B levels are a good prognostic marker for mild traumatic brain injury [38, 39] but S100B levels increase with time. This may be due to the contribution from infiltrating immune cells mainly neutrophils and the activation of inflammatory pathways mediated by DAMPs in the early stage followed by the role of DAMPs and PAMPs in the late stage. These findings warrant further investigation on this aspect to identify better novel markers for severe brain injury.

### 3.3 Nuclear and Mitochondrial DNA

Recognition of nuclear DNA by circulatory monocytes triggers an inflammatory immune response and stimulates the production of inflammatory cytokines including IL-6 and IL-8 [40, 41]. Additionally, mitochondrial damage and release of mitochondrial (mt)DNA, N-formyl peptides (NFPs), mitochondrial transcription factor (TFAM), cardiolipin, and ATP also induce SIRS after injury (Figure 1). The release of these mitochondrial components (mtDAMPs) induces inflammatory response through endosomal localized TLR9, cytosolic cyclic GMP-AMP synthase (cGAS)-STING or NLRP3 inflammasome activating nuclear factor-kappa beta (NF-κB), caspase-1, and stimulator of interferon genes (STING) and increasing the secretion of IL-6, TNF-α, IL-1β, IL-18, and interferon (IFN)-1. The release of proinflammatory cytokines IL-6, IL-8, and IL-1β from monocytes is also mediated by the release of mtDAMPs (Figure 1). The inflammatory response mediated by excessive mtDAMPs leads to MODS and poor clinical outcomes [42–44]. Since mtDAMPs play a critical role in SIRS, mtDAMPs may be used as biomarkers to evaluate the trauma severity and decide on treatment strategies. This notion is also supported by the fact that systemic administration of mtDNA induces a strong inflammatory response [45]. In addition to mtDNA from mitochondria, other mtDAMPs like mitochondrial formyl peptides (mtFP), heme signaling, inflammatory purinergic signaling by ATP, cardiolipin, cytochrome C, and transcription factor A of mitochondria (TFAM) also play a critical role in trauma and post-injury [46, 47]. Another study by Aswani et al. reported that the release of mtDNA after trauma is sufficient for inducing an immune response and the concentration of mtDNA after injury depends on the severity of trauma and the time of sampling after trauma. Further, in-vivo results showed that nucleic acid scavenging polymer, hexadimethrine bromide (HDMBr), reduces the severity of organ injury by scavenging circulating mtDNA and nuclear DNA [48]. Thus, HDMBr could have therapeutic potential in trauma-induced MODS in human patients to improve clinical outcomes.

### 3.4 Heat Shock Proteins

Levels of heat-shock proteins (HSPs) are increased in the circulation/ extracellular compartment after trauma, and thus may serve as a biomarker during the early stage for trauma severity. Increased levels of HSP72 have been reported in severe trauma patients. HSPs are involved in the immune response by promoting antigen presentation, dendritic cell maturation, and upregulation of major histocompatibility complex (MHC) class II molecules, CD80, and CD86 [8]. The role of HSPs in inducing inflammation in severe trauma patients is also suggested by significantly increased serum concentrations of HSP27 and HSP70 in severely injured polytrauma patients and their association with poor prognosis [49, 50]. The study also concluded that thoracic trauma in polytrauma patients further increases HSP27 and HSP70 levels concomitantly. After severe trauma, inflammatory cytokines IL-6, TNF-α, and IL-1β are involved in the inflammatory response needed for repair but a hyperinflammatory state causes MODS, immunosuppression, and release of HSPs. HSPs acting as DAMPs further deteriorate the immune function. HSPA1A is released in large quantities after severe polytrauma, and the levels increase more in MODS, thus may serve as a diagnostic and prognostic biomarker [51]. HSPs play a role in immunosuppression in the later stage but their levels increased early in trauma as early as 30 minutes after polytrauma and significantly increased HSP72 levels were found in patients with polytrauma [52]. Though higher HSP72 levels were associated with patient survival after severe trauma but not with incidence or severity of the post-injury inflammatory response or organ dysfunction [52]. Regarding the mechanism of action of HSPs, Liu et al. [53] reported that HSPs signal through NF-κB and mediate oxidative stress-induced inflammation. These results suggest that HSPs play a critical role in post-trauma inflammation (Figure 1) and HSP70, HSP-27, SHP-72, and HSPA1A may be used as biomarkers in severely injured polytrauma patients.

### 3.5 NETs

In addition to mediating inflammatory immune response through its downstream signaling, DAMPs may also mediate inflammatory response by inducing neutrophil extracellular traps (NETs), released from neutrophils during inflammation. NETs, comprised of extracellular DNA with histones, myeloperoxidase, and elastase, basically capture and eliminate pathogens. However, excessive formation of NETs may mediate exaggerated inflammatory immune responses perpetuating the release of inflammatory cytokines. This may contribute to a cascade of DAMPs-NETs-cytokines mediated persistent inflammation, sepsis, MODS, and death in polytrauma patients [54, 55] and thus DAMPs-induced NETs activation (Figure 1) should be an interesting area of research to improve clinical outcomes in polytrauma patients. This notion is further supported by the findings of NETs as one potential source of circulating cell-free DNA (cfDNA) which contributes to post-trauma inflammation and coagulation regulation [56]. It is important to note that cfDNA has a different source of secretion. Chornenki et al. [56] reported that the source and mechanism of release of circulating cell-free DNA (cfDNA) are different for trauma and sepsis patients and cfDNA is mainly released from activated neutrophils via NETosis in sepsis while from injured and necrotic tissue in trauma. Further, Stortz et al. [57] reported that it is cell-free (cf) nuclear DNA (ncDNA) but not mtDNA concentrations significantly correlate with the early inflammatory response after severe trauma. Additionally, the study also reported that IL-6 and leukocyte transcriptomics are better predictors for the clinical outcome, not cfDNA and mtDNA are not associated with adverse prognosis. Based on these contradictory findings it is important to further investigate the role of mtDNA in the prognosis and stratification in severe polytrauma patients. Overall, DAMPs released after trauma not only induce an innate immune and inflammatory response, as discussed in [11], but are also involved in inducing immunosuppression and rendering patients prone to infection with the involvement of HSP70, nuclear DNA, and HLA-DR [58], endotoxin tolerance, and epigenetic alterations increasing susceptibility to secondary infections [8].

In the early stage of trauma, DAMPs mediate SIRS-mediated multiorgan dysfunctional syndrome (MODS) and altered coagulation while in later phases DAMPs are involved in nosocomial infection and sepsis [8, 13]. Thus, targeting DAMPs using specific antibodies and small molecules may have therapeutic efficacy, and amelioration of sepsis and MODS in animal models support the notion of targeting DAMPs [59]. The positive results in animal models indicate that targeting DAMPs in the early phase of injury may prevent persistent inflammatory responses and organ failure. In the context of trauma, HMGB-1 is the most studied DAMP and there is a need to investigate the role of other DAMPs in the pathophysiology as well as their role as a biomarker in trauma patients. It should also be noted that the plasma profile of different DAMPs may differ in different types of trauma like burns and hemorrhagic shock [60, 61], thermal injury [62, 63], road traffic accidents/major trauma [20, 21], and explosion/blast injury [64]. The differential expression of various DAMPs in different types of injuries and their changing levels with time is an important topic to be critically examined.

## 4. Trauma, PAMPs, and Inflammation

Along with DAMPs, PAMPs also contribute to inducing inflammatory response by increased secretion of pro-inflammatory cytokines and may also do the same synergistically with DAMPs, as discussed above, in the later stage in mediating sepsis. However, the question is whether in between DAMPs and PAMPs, which one induces a stronger inflammatory response or is there any differential response in terms of the effect on ongoing various pathologies and strength? Eppensteiner et al. [65] reported that DAMPs, compared to PAMPs, induce weaker immune responses, less TLR signal desensitization, and less innate immune cell death but stronger systemic coagulopathic effects. Though DAMPs and PAMPs have differential effects on pathogenesis, both contribute to systemic immune response, MODS, and late mortality in patients with a critical illness. The study also proposed that it is not the expression levels/volume of DAMPs, but the activity levels that may be more important to target to achieve a better therapeutic outcome. Since DAMPs and PAMPs are secreted after polytrauma, these findings may have implications while evaluating the biomarkers in the intensive care unit for diagnostic decision-making early after an injury.

PAMPs like bacterial endotoxins, in addition to TLRs, can also activate stress response gene heme oxygenase-1 (HO-1) and nrf2 which can further increase the amount of another DAMP ATP perpetuating PAMP-DAMP-inflammatory cascade and may lead to chronic inflammation and organ damage after trauma [14]. Heme binds to TLRs and increases the secretion of IL-1β and HO-1 through activation of NF-κB. In the presence of bacteria, heme will increase ATP production causing increased conversion of pro-IL-1β to active IL-1β. This cascade results in persistent inflammation and may cause MODS. LPS is the main endotoxin released from bacteria and induces SIRS involving induction of inflammatory response, oxidative stress, and protein synthesis causing MODS [66]. Among the signaling pathways, LPS signals through TLRs and induce inflammation involving MyD88, TIRAP, TRIF, IRAK1, IRAK4, TRAF6, NF-κB, MAPKs (p38, JNK, and ERK1/2), and IRF3 and increase secretion of inflammatory cytokines, chemokines, and interferons [67, 68]. Since inflammatory mediators involved in LPS signaling are also secreted after trauma and are involved in DAMPs signaling, in the late stages of severe trauma, the synergistic action of DAMPs and PAMPs mediate more detrimental effects and thus therapeutic strategies should be designed targeting both DAMPs and PAMPs.

## 5. Trauma, Pattern Recognition Receptors (PRRs), and Inflammation

Pattern recognition receptors comprise TLRs, nucleotide-binding and oligomerization domain (NOD)- like receptors (NLRs), C- type lectin receptors (CLRs), a retinoic acid-inducible gene I (RIG- I)-like receptors (RLRs), RAGE, and various intracellular DNA sensors. Recognition of LPS by TLRs activates downstream signaling and induces secretion of pro-inflammatory cytokines mediating inflammatory response [69, 70]. As discussed above, TLR-2, -3, -4, and -x9 are activated by HMGB-1 and LPS and induce an inflammatory response. It should be noted that HMGB-1 does not directly activate TLRs, but HMGB-1 is internalized into macrophages through class A scavenger receptors. Among M1 and M2 macrophages, pro-inflammatory M1 macrophages secrete cytokines in response to HMGB1 but not the anti-inflammatory M2 phenotype [71]. The study suggested that class A scavenger receptors act as co-receptors of HMGB1 for TLR activation. These results suggest that the effect of HMGB-1-mediated TLR activation can be attenuated by targeting the class A scavenger receptor. This is important because no study has investigated the effects of targeting co-receptor to mitigate the effects of TLRs activation. However, TLRs activation through LPS does not involve this mechanism.

## 6. Biomarkers

Inflammation plays a critical role post-trauma in both clearing the wound of debris and wound healing. Thus, inflammatory mediators may play an important role in early diagnosis and plasma levels of cytokines like IL-1, IL-2, IL-6, IL-12, IL-8, IL-4, IL-10, IL-17, IL-13, TNF-α, IFN-γ, and TGF-β, complement C3a and C5a, leukotrienes, prostaglandins, thromboxane, and immune cells might be diagnostic as well as prognostic [72] for SIRS. Additionally, the levels of DAMPs, PAMPs, and PRRs may also be diagnostic as they play a critical role in the pathogenesis of MODS in polytrauma as discussed above. However, these mediators are also elevated during sepsis [73], and thus lessen the diagnostic and prognostic value of these mediators for SIRS and differentiation between SIRS and sepsis. Thus, it is important to distinguish between SIRS and sepsis and stratify the injury or sepsis based on the biomarkers specific to organs in the body [73] and the timing of sample collection after trauma as discussed below, and this might be helpful because sepsis occurs late after trauma during immunosuppression.

Along with the existing pro- and anti-inflammatory biomarkers, other biomarkers such as MMPs and selectins (to differentiate between SIRS and sepsis), IL-1α, IP-10, sTNF-R2, and sFas (indicating the progression of sepsis to shock), markers specific for signaling pathways and specific to an organ injury might help in discriminating between SIRS and sepsis [73–75]. The role of biomarkers asides from inflammatory biomarkers to improve prognosis by modifying the treatment based on biomarkers levels has been discussed in the literature [76]. Schrijver et al. [77] conducted a study to determine the value of myeloperoxidase as a biomarker for mortality in SIRS and sepsis patients. In a population of patients with trauma-induced inflammation, the study found that MPO levels in patients with sepsis were significantly higher compared to those without sepsis. Results showed an average MPO level of 60 ng/mL in sepsis patients and 43 ng/mL in SIRS patients. A similar study by Sung Cha et al. [78] evaluated the usefulness of the myeloperoxidase index for a differential diagnosis of SIRS. The median myeloperoxidase index was found to be higher in sepsis versus non-infectious SIRS. Findings also showed an increased δ neutrophil index, as well as elevated levels of white blood cells and C-reactive protein. However, in analysis, they found that the myeloperoxidase index was not statistically useful as a diagnostic parameter. A blinded, prospective cohort study by Crousor et al. [79] examined whether analyzing cell volume criteria in addition to the white blood cell count was beneficial in differentiating between patients with SIRS, sepsis, and septic shock. The results established monocyte distribution width (MDW) as a parameter to identify sepsis from other types of trauma-induced inflammation. Additionally, findings showed a positive correlation between MDW and infection severity.

A prospective observational study [80] of ICU patients of the University Hospitals of Lille, France, and Geneva, Switzerland, evaluated the proteoglycan, endocan, as a diagnostic and prognostic biomarker for sepsis. Circulating levels of endocan were found to be significantly elevated in sepsis (1.9 ng/mL), severe sepsis (1.97 ng/mL), and septic shock (6.11 ng/mL) compared to systemic inflammatory response syndrome (0.72 ng/mL). The role of endocans as a biomarker for sepsis is further supported by the findings of Mihajlovic et al. [81] concluding that endothelial biomarkers have a good diagnostic and prognostic potential for sepsis and may predict the severity and fatality of sepsis. Further, an ANOVA analysis by Punyadeera et al. [75] showed that the levels of MMP-1, -2,-7, -13, and E selectin were significantly higher in SIRS cases compared to septic cases. The study also found higher serum levels of IL-1α, IP-10, and sTNF-R2 in septic patients compared to SIRS patients and elevated values of IP-10, sFas, and sTNF-R2 in patients in septic shock.

Based on these studies, it is obvious that there is a need to have more specific biomarkers to differentiate between SIRS and sepsis and newer biomarkers may play a role (Table 1). Further, there is also a need for future research considering the factors affecting SIRS and sepsis, the effect of mediators secreted as a response to acute inflammation-anti-inflammatory mediators on the levels of inflammatory mediators, and standard definitions for the level of biomarkers and staging criteria for interpretation of the data, their prognostic and diagnostic values, their sensitivity and specificity and thus, large scale well planned clinical trials are warranted [82, 83]. In addition to the markers related to inflammation and involved in DAMPs and PAMPs signaling, epigenetic biomarkers mainly microRNA are an upcoming area of research and should be considered [84–87]. Although discussing the role of miRNAs is out of the scope of this review, it is important to mention the role of miRNAs because the expression of various pro- and anti-inflammatory mediators is regulated by miRNAs, and changing miRNAs after trauma may affect the expression levels of various biomarkers.

The studies highlighted in this section identify potential biomarkers that may be applied to improve patient outcomes in the future. However, there still appears to be limited research into biomarkers that identify different types of trauma-induced inflammation. Further research is needed to identify these markers and evaluate their diagnostic value. Considering the timing of sample collection, the type of sample, type of trauma, relating and interpreting the levels of biomarkers in the context of organ injury, and combining biochemical biomarkers with metabolomics and proteomics, as discussed in the next section, might have more diagnostic and prognostic value.

## 7. Type of Sample

To assess the severity, and stratification of the polytrauma patient, and for prognostic evaluation, blood plasma levels of various biomarkers, as discussed above, are commonly used. Serum levels of S100B are a good biomarker and prognostic indicator for traumatic brain injury [88]. However, studies reported that other body fluids can also be used for evaluating the biomarkers and they also have prognostic and monitoring values. For example, salivary S100B has been reported as a biomarker for repeated head injuries causing concussions in water polo athletes and S100B levels are prognostic of repeated head impacts causing axonal injury even in asymptomatic athletes [89]. However, Hasselblatt et al. [90] reported that increased serum levels in sprinters might originate from extracranial sources and are not associated with brain injury. These two studies examined S100B levels from two different sources and have different conclusions. Moreover, a recent metabolomic study on 716 patients with traumatic brain injury concluded that a simple blood sample is indicative of brain injury severity [61]. Though this study evaluated the changes at metabolomics levels and not the levels of DAMPs and PAMPs, whether a combination of biochemical and metabolomics will have more predictive and prognostic value remain a topic to elaborate on and investigate.

Urine analysis is a commonly used investigation for assessing renal function during illness and kidney injury during trauma, however, Xie et al. [91] reported that both urine and blood have diagnostic and prognostic significance in sepsis-associated acute kidney injury. The study reported elevated levels of urine and serum neutrophil gelatinase-related lipid carrier protein (NGAL), urinary IL-18, Kim-1, Netrin-1, sCD163, and serum estradiol and serum soluble thrombolytic regulatory protein in sepsis-associated acute kidney injury and concluded urinary Kim-1 > urinary NGAL > blood NGAL > urinary IL-18 as a sequence for diagnosis. These results suggest that a combination panel of both urinary and blood biomarkers is more beneficial and has differential but higher prognostic value. These results became more important in the light of a report from a study on dogs reporting that moderate sensitivities and specificities may reduce the predictive value of individual urinary biomarkers [92]. Further, the findings of elevated plasma mtDNA concentrations in non-infectious SIRS but not correlate with biomarkers of systemic inflammation or renal injury. Moreover, the elevation of mtDNA plasma levels in critical illnesses, including sepsis, trauma, and cardiac arrest but no correlation with inflammation, immune dysfunction, and organ damage biomarkers suggests that plasma mtDNA does not play a role in these pathologies. However, mtDNA has a prognostic value for kidney injury. These findings are indicative of prognostic value for plasma and urinary mtDNA but the cause-effect relation of mtDNA in severe injury and kidney injury remains topic of discussion and investigation [58, 93, 94].

## 8. Timing of Assessment and Clinical Significance

### 8.1 Importance of Timing

As discussed above, DAMPs and PAMPs play a crucial role in the pathogenesis of SIRS and sepsis through PRRs in the short and long term, thus the timing of assessing a trauma patient for these as biomarkers and analysis and interpretation of the levels of various markers including HMGB-1, S100 proteins, TLRs, RAGE, sRAGE, HSPs, HLA-DRs, and cytokines is of utmost importance. This notion is supported by the finding of changing cell surface receptors on CD4+ (increased expression of PD-1), CD4+ and CD8+ T-cells (decreased expression of B- and T-lymphocyte attenuator), and of TLRs on CD14+ monocytes after 6 months and decreased secretion of IL-6 and TNF-α [95]. The importance of timing to collect the samples for assessment and as a biomarker is also supported by the results of changes in serum S100B concentration during the first day of injury. The study reported that S100B, a biomarker of brain injury, peaks at 27.2 hours and then declines [96]. The authors concluded that even a small difference in injury to sample collection time may lead to marked changes in S100B concentration and thus the timing of sample collection must be considered while interpreting results. The importance of early sampling for biomarkers was also reported by changing expression levels of S100B after traumatic brain injury and the suggestion of collecting samples early after trauma [31]. These findings suggest the importance of a time-based strategy for the stratification of polytrauma patients.

The importance of assessing polytrauma patients and interpreting the levels of the biomarkers with time has been conceptualized since the levels of cytokines, chemokines, DAPs, PAMPs, PRRs, histones, nuclear DNA, mtDNA may change with time, and thus, while treating polytrauma patients, treatment strategies should incorporate time of sample collection to assess biomarker and scoring using other criteria for a better clinical outcome [97]. The importance of including time, a crucial factor in polytrauma patients, while designing treatment is further supported by the findings of higher MODS and SIRS scores with increased mortality and an increase in these scores with time in non-survivors while a decrease in subjects with positive outcome [98]. Further, different peaks of different types of mtDAMPs suggest the importance of timing while collecting samples and interpreting data [48]. Moreover, the assessment of various mediators like cytokines (TNFα, IL-6, IL-10), HMGB-1, and adhesion molecule (ICAM-1) with time should be time bound and accordingly the severity and outcome be decided. This is because the peak of each cytokine may differ with time and its association as increased IL-6 at the time of admission is associated with injury severity score, IL-10 with SIRS with hypoperfusion, and HMGB-1 with shock. At 72 hours, increased IL-6 and IL-10 levels are associated with MODS and death while low TNFα/IL-10 and IL-6/IL-10 ratios at 24 and 72 hours are associated with MODS and death [99]. These findings suggest that each biomarker should be assessed independently as well as in correlation with others at different time points. This will help in stratifying the patients as per severity, deciding treatment strategies in a time-bound manner, and increasing clinical outcomes.

### 8.1 Translational and Clinical Significance

DAMPs are secreted after trauma and levels of DAMPs may have clinical significance to predict the outcome and decide the future course of action and treatment strategies. Matsumoto et al. [100] reported that compared to control, patients with sepsis had significantly increased levels of soluble (s) RAGE and were significantly associated with Acute Physiology and Chronic Health Evaluation II (APACHE II), Sequential Organ Failure Assessment (SOFA), and International Society of Thrombosis and Haemostasis (ISTH) overt disseminated intravascular coagulation (DIC) scores. The study also reported that increased levels of sRAGE were also correlated with the upregulated IL-6, soluble vascular adhesion molecule (VCAM) 1, and plasminogen activator inhibitor (PAI) 1 level and a reduction in platelet count. The study concluded that increasing levels of sRAGE positively correlate with progression of DIC and severity of sepsis, signifying severity of inflammation, endothelial cell injury, and alteration in the coagulation cascade. These findings suggest the importance of sRAGE as a biomarker in sepsis and may also have implications in trauma patients in both early and late stages as DAMPs are secreted in the early stage and have synergistic action with PAMPs in mediating MODS and sepsis, as discussed above, in the late stage after trauma.

Since SIRS is an early pathological process and sepsis is a late pathological outcome after a polytrauma affecting the clinical outcome, it is very important to distinguish between sepsis and SIRS. No single biomarker can distinguish between these two, however, a panel of biomarkers may be used to distinguish between these two pathologies. Cahil et al. [101] performed multiplex plasma immune mediator signature and reported that the cytokines levels entirely differ between SIRS and sepsis and infection significantly increases the level of IL-6, IL-1α, and TREM-1 while injury suppresses the levels of MDC (C-C motif chemokine 22), TREM-1, IP-10 (C-X-C motif chemokine ligand 10 also known as Interferon gamma-induced protein 10), MCP-3 (monocyte chemoattractant protein 3), FLT3L (Fms Related Receptor Tyrosine Kinase 3 Ligand), Tweak (tumor necrosis factor-like weak inducer of apoptosis), GRO-α (interleukin-8-related chemotactic cytokine), and ENA-78 (C-X-C motif chemokine 5). APACHE II, SOFA, and ISTH-DIC scores are various strategies to evaluate a patient in emergency settings after a polytrauma, but the question is whether one scoring criteria is enough or whether we should evaluate the patient with a combination of different criteria. Liu et al. in a meta-analysis reported no significant differences in the accuracy of diagnosis of sepsis between positive quick SOFA scores and SIRS criteria and concluded that a combination of qSOFA and SIRS scoring will have better prognostic value in predicting mortality compared to anyone alone [102].

## 9. Conclusion

Stratification of patients after polytrauma is important to enhance prognosis and clinical outcome and biomarkers play a critical role during the assessment. Based on the studies discussed above, a time-bound assessment and interpretation of the biological samples are important. Additionally, the clinicians in an emergency should also focus on the type of sample and analysis because along with the biochemical analysis, other investigations including metabolomics, proteomics, and microarray may have an additive value in planning treatment strategies for a better outcome. Moreover, the diagnosis of organ injury depending on time may also be sample specific. These aspects are critical but have not been fully investigated and warrant well-planned large-scale studies. Finally, it is important to distinguish SIRS from sepsis because many biomarkers are the same and the time factor plays a critical role in the switch from SIRS to sepsis.

## Figures and Tables

**Table 1:** Biomarker for the Differentiation of SIRS and Sepsis.

Biomarker(s)	Type of Study	Aim of the Study	Results	Citation
Myeloperoxidase (MPO)	Observational, Single Center Cohort Study	Determine the value of MPO as a biomarker for mortality in SIRS and sepsis patients in the ICU	In a population of SIRS patients, MPO levels in patients with sepsis were significantly higher compared to those without Sepsis with an average MPO of 60 ng/mL versus 43 ng/mL.	[77, 78]
Monocyte Distribution Width (MDW)	Blinded, prospective Cohort Study	Determine if volume increases of circulating immune cells add value to the white blood cell count for early septic detention in the ED	Crousor et al. established monocyte distribution width (MDW) as a parameter to differentiate between sepsis from systemic inflammatory response syndrome (SIRS) and infection.	[79]
Endocan	Prospective Observational Study	Evaluate serum levels of endocan in septic patients and determine its potential as a diagnostic or prognostic marker of sepsis.	Circulating levels of endocan were found to be significantly elevated in sepsis (1.9 ng/mL) severe sepsis (1.97 ng/mL), and septic shock (6.11 ng/mL) compared to systemic inflammatory response syndrome (0.72 ng/mL).	[80]
IL-1α, IP-10, and sTNF-R2	Observational Study	Identify biomarkers for the differential diagnosis of SIRS versus sepsis, and the various stages of sepsis.	Serum levels of IL-1α, IP-10, and sTNF-R2 were higher in sepsis, severe sepsis, and septic shock compared to SIRS	[75]
MMP-1, -2, -7 and -13	Observational Study	Identify biomarkers for the differential diagnosis of SIRS versus sepsis, and the various stages of sepsis.	MMP-1, -2, -7 and -13 plasma concentrations showed to be significantly higher in SIRS patients when compared to those with sepsis.	[75]
sE-selectin	Observational Study	Identify biomarkers for the differential diagnosis of SIRS versus sepsis, and the various stages of sepsis.	Soluble E-selectin concentrations showed to be significantly higher in SIRS patients when compared to those with sepsis.	[75]
